# Temporal Dynamics of the Association Between Acute Kidney Injury and Mortality After Transcatheter Aortic Valve Implantation: Insights from Time-Varying and Landmark Survival Analyses

**DOI:** 10.3390/jcdd12120470

**Published:** 2025-11-30

**Authors:** Seda Elcim Yildirim, Bahadır Akar, Berkay Palac, Hakan Bozkurt, Tarik Yildirim, Tuncay Kiris, Eyüp Avci

**Affiliations:** 1 Department of Cardiology, School of Medicine, Balikesir University, 10900 Balikesir, Turkey; 2 Department of Cardiology, Atatürk Training and Research Hospital, Izmir Katip Çelebi University, 35360 Izmir, Turkey

**Keywords:** transcatheter aortic valve implantation, acute kidney injury, time-varying Cox regression, all-cause mortality

## Abstract

Background: Acute kidney injury (AKI) is a frequent complication following transcatheter aortic valve implantation (TAVI) and has been linked to increased mortality. However, the temporal pattern of this association remains uncertain. This study aimed to evaluate the time-dependent impact of AKI on mortality after TAVI using advanced survival analyses. Methods: We retrospectively analyzed 381 consecutive patients who underwent transfemoral TAVI between December 2016 and October 2024 at two tertiary cardiovascular centers. AKI was defined according to the Acute Kidney Injury Network (AKIN) criteria. The primary outcome was all-cause mortality. Patients were categorized into AKI and non-AKI groups. Clinical outcomes, including 30-day, 1-year, and overall mortality, were evaluated. Results: Among 381 patients who underwent TAVI, 59 (15.5%) developed AKI according to the AKIN criteria. During a 33.9 months (18.0–59.2) median follow-up of overall mortality was significantly higher in the AKI group compared with those without AKI. In the multivariate Cox regression analysis, AKI was significantly associated with long-term mortality (HR: 2.07, 95% CI 1.32–3.25; *p* = 0.002). The time-varying hazard ratio curve demonstrated that the excess mortality risk associated with AKI was most pronounced in the early period and gradually declined thereafter. In time-interval–specific analyses, AKI was strongly associated with mortality within the first month (HR 6.30, 95% CI 3.03–13.08, *p* < 0.001) and remained significant up to 12 months (HR 2.18, 95% CI 1.32–3.59, *p* = 0.002). Beyond the first year, this association attenuated and lost statistical significance at 12–36 months (HR 0.90, *p* = 0.79), 36–60 months (HR 0.57, *p* = 0.24), and >60 months (HR 0.43, *p* = 0.13). Conclusions: AKI is an important predictor of early and mid-term mortality following TAVI, but its long-term prognostic impact is less pronounced. Preventive strategies and early management of AKI may improve outcomes in this high-risk population.

## 1. Introduction

Acute kidney injury (AKI) is a well-recognized complication following the use of iodinated contrast media in various cardiovascular interventions [1,2]. In the setting of transcatheter aortic valve implantation (TAVI), the occurrence of AKI is of particular concern, as these patients are typically elderly, frail, and burdened with multiple comorbidities such as chronic kidney disease, diabetes mellitus, and heart failure. The incidence of AKI after TAVI has been reported to range between 10 and 30%, depending on the definition used and baseline renal function [3,4,5]. Even though TAVI is a less invasive alternative to surgical aortic valve replacement, the exposure to contrast medium during pre-procedural imaging and the intervention itself places patients at a considerable risk of renal injury.

Numerous studies have demonstrated that AKI is associated with increased in-hospital mortality, longer hospital stay, and higher rates of major adverse cardiovascular events [6,7,8]. However, most of these investigations have primarily focused on the acute and peri-procedural consequences of AKI, whereas its long-term prognostic implications remain less clearly defined. Importantly, the effect of AKI on mortality may not be constant over time. While the early hazard is likely driven by acute renal dysfunction, volume overload, and hemodynamic instability, the long-term impact may be attenuated by competing risks and the influence of other comorbidities [9,10].

To address these gaps, advanced survival methodologies such as landmark analysis and time-varying Cox regression are required. Landmark analysis allows the separation of early and late effects, while time-varying Cox models provide insight into how the hazard ratio of AKI evolves over time. In this study, we aimed to evaluate the prognostic significance of AKI in patients undergoing TAVI, focusing on its temporal impact on all-cause mortality. By combining landmark analysis at 30 days and 12 months with time-varying Cox regression, we sought to provide a comprehensive assessment of the dynamic nature of AKI’s prognostic role.

## 2. Methods

This retrospective study included 381 consecutive patients who underwent transfemoral TAVI between December 2016 and October 2024 at two tertiary cardiovascular centers. Patients were eligible for inclusion if baseline and post-procedural serum creatinine values were available to determine AKI according to the Acute Kidney Injury Network (AKIN) criteria [11]. Stage 1: increase in serum creatinine (SCr) of 150–199% (1.5–1.99× increase compared with baseline) or increase of ≥0.3 mg/dL (≥26.4 mmol/L) or urine output (UO) < 0.5 mL/kg/h for >6 h but <12 h; Stage 2: increase in SCr of 200–299% (2.0–2.99× increase compared with baseline) or UO < 0.5 mL/kg/h for >12 h but <24 h; Stage 3: increase in SCr of ≥300% (>3× increase compared with baseline) or SCr of ≥4.0 mg/dL (≥354 mmol/L) with an acute increase of at least 0.5 mg/dL (44 mmol/L) or UO < 0.3 mL/kg/h for ≥24 h or anuria for ≥12 h or renal replacement therapy administration (irrespective of other criteria). Chronic kidney disease (CKD) stages were assigned according to the KDIGO classification [12] based on baseline estimated glomerular filtration rate (eGFR). Patients were categorized as follows: CKD stage 1: eGFR ≥ 90 mL/min/1.73 m^2^, 00CKD stage 2: eGFR 60–89, CKD stage 3a: eGFR 45–59, CKD stage 3b: eGFR 30–44, CKD stage 4: eGFR 15–29, CKD stage 5: eGFR < 15 (not on dialysis).

The following exclusion criteria were applied to ensure homogeneity and data reliability: End-stage renal disease requiring chronic dialysis or baseline estimated glomerular filtration rate (eGFR) < 20 mL/min/1.73 m^2^ prior to the procedure, missing or incomplete serum creatinine data within the first 48–72 h post-procedure, precluding accurate AKI assessment, history of prior transcatheter or surgical aortic valve intervention, conversion to open-heart surgery during TAVI or periprocedural death within 24 h, active infection, sepsis, or severe hemodynamic instability before the procedure, periprocedural administration of nephrotoxic agents (e.g., aminoglycosides, amphotericin B, cisplatin) that could independently induce renal injury, inadequate follow-up data or loss to follow-up for mortality assessment, missing essential baseline clinical or laboratory data, including diabetes status, renal function, or contrast volume information. After applying these criteria, a total of 381 patients were included in the final analysis. Data were extracted from the hospital’s electronic medical records and included demographic characteristics, clinical parameters, and laboratory values. While using R STUDIO 2025 version 4.5.1 (Vienna, Austria) commands and some synthesis, we received help from GenAI tools.

## 3. Procedure

TAVI was performed using self- or balloon-expandable prostheses. All procedures were carried out in accordance with established techniques. The choice of anesthesia type and vascular access route was left to the discretion of the operating team. The transfemoral approach was preferred as the first-line access.

### 3.1. Study Endpoints

The primary outcome was long-term mortality, defined as death from any cause during the follow-up period.

### 3.2. Statistical Analysis

Continuous variables were presented as mean ± standard deviation (SD) and compared using Student’s *t*-test or Wilcoxon rank-sum test, as appropriate. Categorical variables were summarized as frequencies and percentages and compared using Pearson’s chi-squared or Fisher’s exact test.

To evaluate the dynamic effect of AKI on all-cause mortality following TAVI, a time-varying Cox proportional hazards regression model was fitted. The time-varying hazard ratio [HR(t)] was estimated across the follow-up period using flexible modeling with splines, and visualized with 95% confidence intervals (CI). This model enabled the detection of changing relative risk over time, capturing the early impact and potential late attenuation of AKI’s prognostic effect. Landmark analyses were performed at predefined time points (0–1 month, 1–12 months, and >12 months) to assess the temporal association between AKI and mortality within clinically meaningful intervals. Separate Kaplan–Meier survival curves were constructed for each interval, and survival differences were compared using the log-rank test. This approach mitigated immortal time bias and allowed for interval-specific survival interpretation. To explore effect modification, a series of stratified Cox proportional hazards models was conducted across clinically relevant subgroups (e.g., age, baseline eGFR, Chronic Obstructive Pulmonary Disease [COPD], valve type, mitral/tricuspid regurgitation, hemoglobin levels). Hazard ratios with 95% CIs were reported within each subgroup, and statistical interaction was tested using multiplicative interaction terms. A forest plot was generated to visualize heterogeneity of AKI-related risk across subgroups. Differences in cumulative survival time between AKI and non-AKI groups were further quantified using restricted mean survival time analysis (RMST). RMSTs were calculated at 12, 24, and 60 months, with between-group differences reported alongside 95% CIs. This method provides a clinically interpretable measure of average survival benefit or loss over fixed follow-up durations.

Discrimination performance of the multivariable clinical model, with and without the inclusion of AKI, was assessed using both conventional (static) and time-dependent receiver operating characteristic (ROC) curve analyses. The area under the curve (AUC) was calculated for the overall follow-up (conventional ROC) and at multiple time points (12, 24, 60 months) using time-dependent ROC methods. ΔAUC values and associated *p*-values were reported to determine the incremental predictive value of adding AKI to the base model across time. Confidence bands were calculated using bootstrapping or inverse probability weighting as appropriate. All statistical analyses were conducted using R version 4.5.1 (Vienna, Austria) and SPSS version 30.0 (Armonk, NY, USA), with a two-tailed *p*-value < 0.05 considered statistically significant.

## 4. Results

A total of 381 patients who underwent TAVI were analyzed, of whom 59 (15.5%) developed AKI, while 322 (84.5%) did not. The median follow-up time was 33.9 months (18.0–59.2). The mean age was similar between the AKI and non-AKI groups (77.29 ± 7.09 vs. 77.21 ± 7.24 years, *p* = 0.942). However, gender distribution differed significantly, with a lower proportion of males in the AKI group (36% vs. 51%, *p* = 0.034).

The rates of diabetes mellitus, hypertension, and smoking did not differ significantly between groups (Table 1). There were no statistically significant differences in the prevalence of coronary artery disease, peripheral artery disease, heart failure, COPD, or previous stroke (Table 1).

Platelet count and hemoglobin levels were similar between groups, with no significant differences observed (*p* = 0.983 and *p* = 0.116, respectively). Left ventricular ejection fraction (LVEF) was slightly lower in the AKI group (53.25 ± 11.84% vs. 54.69 ± 10.50%, *p* = 0.344), although not statistically significant.

Notably, the eGFR was significantly lower in the AKI group compared to the non-AKI group (60.14 ± 19.83 vs. 68.87 ± 20.34 mL/min/1.73 m^2^, *p* = 0.003), reflecting compromised baseline renal function. In-hospital mortality was markedly higher among AKI patients (12% vs. 1.9%, *p* = 0.001), and all-cause mortality was also significantly elevated (47% vs. 24%, *p* < 0.001). In total, *n* = 4 patients (1%) required dialysis post-procedure.

There were no significant differences in aortic peak gradient, or mean gradient. However, the rate of moderate to severe mitral regurgitation (51% vs. 37%, *p* = 0.044) and moderate to severe tricuspid regurgitation (41% vs. 25%, *p* = 0.014) were significantly higher in the AKI group, suggesting a more complex or high-risk profile. Valve type distribution showed a trend toward more self-expandable valves in the AKI group (73% vs. 61%, *p* = 0.095), although this was not statistically significant. Pleural effusion was significantly more frequent in the AKI group (56% vs. 35%, *p* = 0.003). Vascular complications were significantly more frequent in patients with AKI compared with those without AKI (*p* = 0.012, Table 1), including higher rates of pseudoaneurysm (5% vs. 1%) and stent-treated arterial injury (9% vs. 3%). In our analysis, red blood cell transfusion rate was significantly higher in the patients with AKI. (18.6% vs. 8.7%; *p* = 0.020, Table 1). According to the KDIGO classification, most patients were in CKD stage 2 at baseline (48.0%). The remaining distribution was as follows: stage 1 in 16.5% (*n* = 63), stage 3a in 17.3% (*n* = 66), stage 3b in 15.5% (*n* = 59), and stage 4 in 2.6% (*n* = 10) of the cohort.

In multivariate analysis; plevral effusion (OR: 2.430, 95% CI: 1.368–4.317, *p* = 0.002) and male gender (OR: 0.465, 95% CI: 0.257–0.841, *p* = 0.011) were independent predictors of AKI.

### 4.1. Comparison Between Survivors and Non-Survivors After TAVI

A total of 381 patients underwent TAVI, of whom 106 (27.8%) died during follow-up, and 275 (72.2%) survived. Non-survivors were significantly older compared to survivors (79.11 ± 7.18 vs. 76.50 ± 7.10 years, *p* = 0.001). Gender distribution was similar between groups, with males comprising 48% in both (Table 2).

COPD was also more common among non-survivors (13% vs. 5%, *p* = 0.007). Additionally, non-survivors had significantly lower hemoglobin levels (10.87 ± 1.87 vs. 11.35 ± 1.71 g/dL, *p* = 0.016), and lower estimated glomerular filtration rate (63.90 ± 22.86 vs. 68.91 ± 19.35 mL/min/1.73 m^2^, *p* = 0.032, Table 2).

Significantly more non-survivors had pleural effusion (54% vs. 33%, *p* < 0.001). Risk indices were elevated in non-survivors, with significantly higher rates of moderate to severe mitral regurgitation (51% vs. 35%, *p* = 0.003) and moderate to severe tricuspid regurgitation (35% vs. 25%, *p* = 0.046). Self-expandable valves were used in 87% of non-survivors compared to 54% of survivors (*p* < 0.001). No significant differences were found in terms of hypertension, diabetes, coronary or peripheral artery disease, atrial fibrillation, or LVEF between survivors and non-survivors. Similarly, peak gradient and mean gradient were comparable between groups (Table 2).

**Figure 1:**
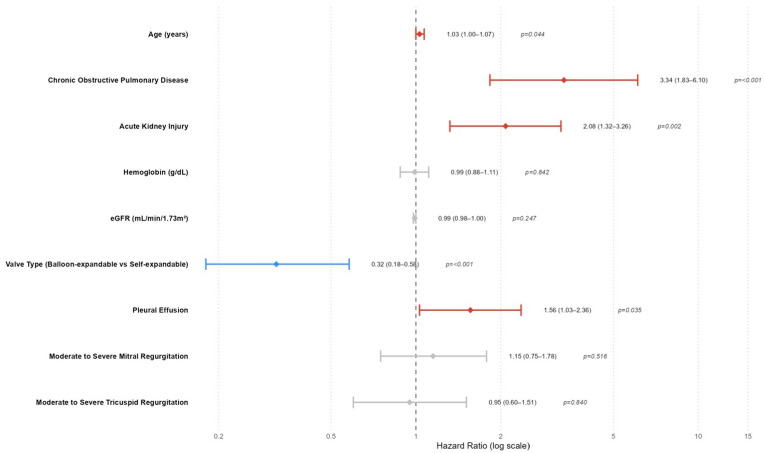
Forest plot of multivariate Cox regressşon analysis for all-cause mortality.

In the Cox regression analysis evaluating predictors of all-cause mortality after TAVI, several variables were found to be significantly associated with all-cause mortality (Table 3, Figure 1). In the multivariate model, age remained a modest but statistically significant predictor of mortality (HR: 1.03; 95% CI: 1.00–1.07; *p* = 0.044), indicating that each additional year of age slightly increased the risk. COPD was a strong independent predictor, with patients exhibiting over a threefold increased risk of mortality (HR: 3.34; 95% CI: 1.83–6.10; *p* < 0.001). Likewise, AKI significantly elevated mortality risk (HR: 2.07, 95% CI 1.32–3.25; *p* = 0.002, Table 3, Figure 1), confirming its critical prognostic value. Pleural effusion also emerged as an independent predictor (HR: 1.56; 95% CI: 1.03–2.36; *p* = 0.035), suggesting that fluid accumulation may reflect or contribute to adverse outcomes. Notably, the type of valve used had a protective effect; balloon-expandable valves were associated with significantly reduced mortality risk compared to self-expandable valves (HR: 0.32; 95% CI: 0.18–0.58; *p* < 0.001). Other variables, such as hemoglobin level, eGFR, and moderate/severe mitral or tricuspid regurgitation, lost significance in the multivariate model despite associations observed in univariate analysis.

**Figure 2:**
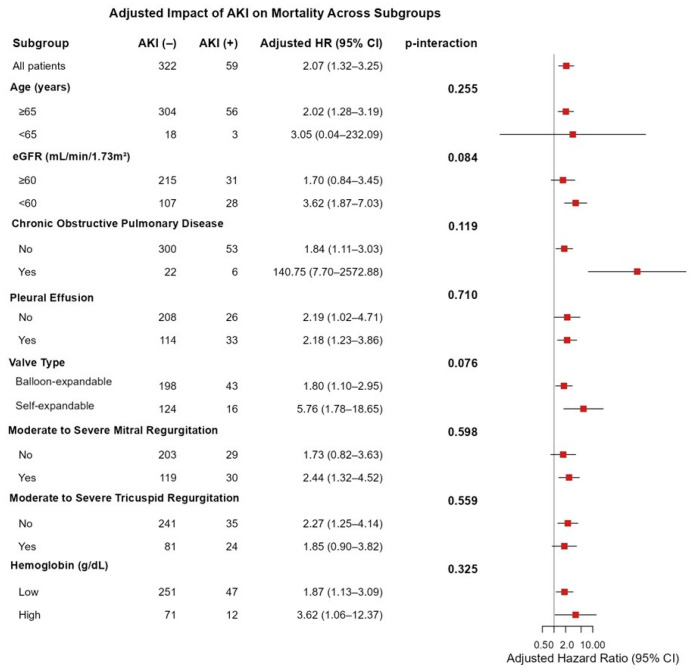
Subgroup analysis of the research cohort utilizing the acute kidney injury (AKI) for the evaluation of all-cause mortality.

In the overall population, AKI was independently associated with increased mortality (adjusted HR: 2.07, 95% CI 1.32–3.25). The subgroup analyses demonstrated that the adverse prognostic effect of AKI on mortality was consistent across all predefined clinical subgroups, with no statistically significant interactions observed (all *p* > 0.05). (Figure 2). Although not reaching statistical significance, the effect of AKI appeared to be more pronounced among patients with reduced renal function (eGFR < 60 mL/min/1.73 m^2^; HR: 3.62, 95% CI 1.87–7.03, p-interaction = 0.084) and those receiving self-expandable valves (HR: 5.76, 95% CI 1.78–18.65, p-interaction = 0.076) (Figure 2).

**Figure 3:**
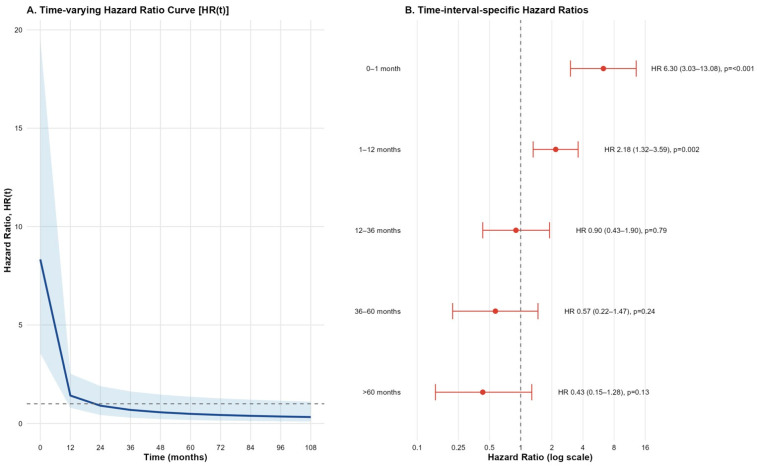
Time-varying hazard ratio (HR) for AKI with 95% confidence intervals.

The time-varying hazard ratio analysis demonstrated that the mortality risk was significantly elevated during the early post-procedural period, with the highest hazard observed within the first month (HR: 6.30; 95% CI: 3.03–13.08; *p* < 0.001, Figure 3), followed by a moderate but significant risk during months 1–12 (HR: 2.18; 95% CI: 1.32–3.59; *p* = 0.002). Beyond 12 months, the hazard ratios decreased substantially and were not statistically significant, suggesting a time-dependent attenuation of mortality risk (Figure 3).

### 4.2. Kaplan–Meier and Landmark Analyses

**Figure 4:**
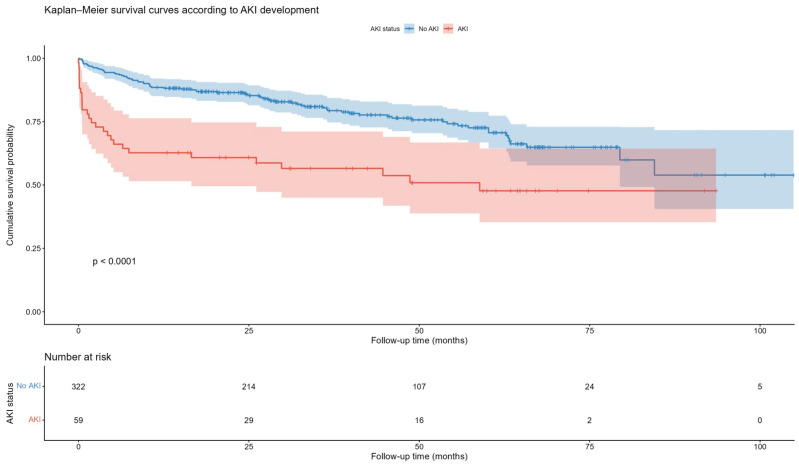
Kaplan–Meier survival curves according to the AKI presence or absence.

Kaplan–Meier survival analysis demonstrated a significantly lower cumulative survival probability in patients who developed AKI following TAVI compared to those without AKI (Log-rank *p* < 0.0001, Figure 4).

**Figure 5:**
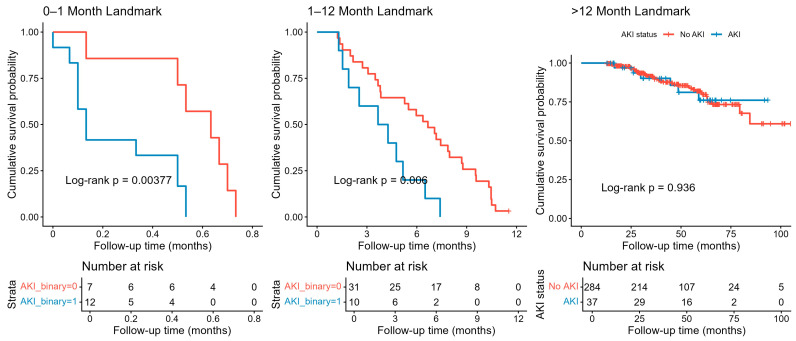
Landmark analysis in the periods of 0–1 month, 1–12 months, and after the 12th month.

Landmark survival analysis revealed a significant impact of AKI on short-term mortality following TAVI. In the 0–1 month interval, patients with AKI exhibited significantly lower survival compared to those without AKI (Log-rank *p* = 0.0038, Figure 5). This difference persisted during the 1–12 month period (Log-rank *p* = 0.006), indicating a continued elevated risk within the first year post-procedure. However, beyond 12 months, survival curves converged, and there was no significant difference between AKI and non-AKI groups (Log-rank *p* = 0.936), suggesting that the impact of AKI on mortality diminishes over time (Figure 5).

### 4.3. Restricted Mean Survival Time (RMST) Analysis

**Figure 6:**
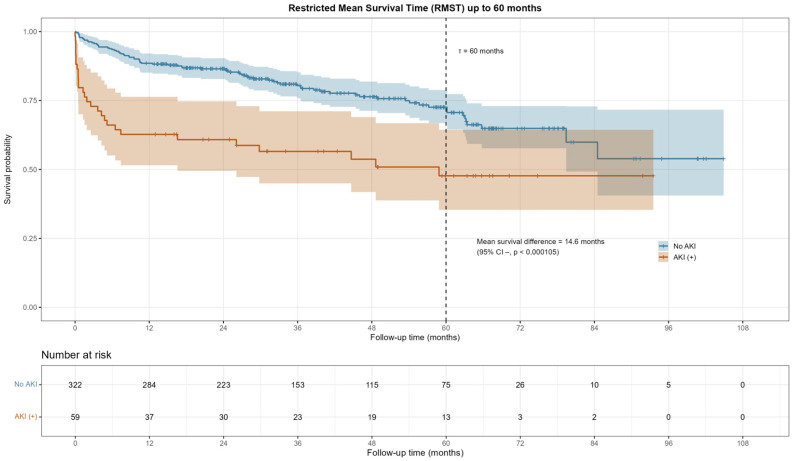
Restricted Mean Survival Time (RMST) Analysis at all times.

**Figure 7:**
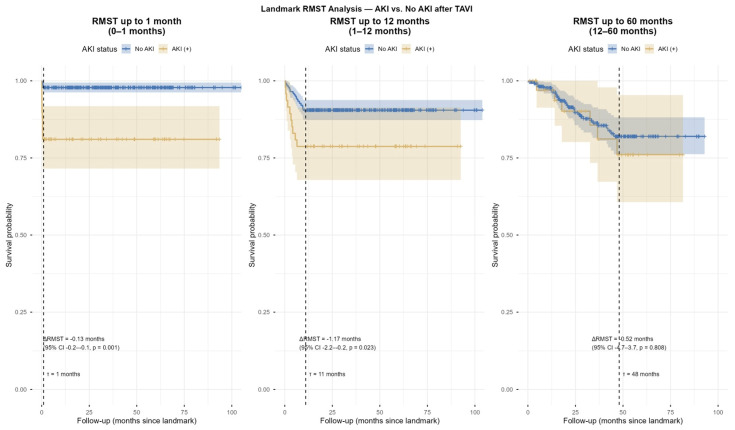
Restricted Mean Survival Time (RMST) analysis in the periods of 0–1 month, 1–12 months, and after the 12th month.

In landmark RMST analysis, patients with post-TAVI-AKI showed significantly reduced mean survival time compared with those without AKI during the early (ΔRMST = −0.13 months, *p* = 0.001) and mid-term (ΔRMST = −1.17 months, *p* = 0.023) periods. However, no significant difference was observed in the long-term follow-up (ΔRMST = 0.52 months, *p* = 0.808, Figure 6 and Figure 7).

### 4.4. Time-Dependent and Conventional AUC Analysis

**Figure 8:**
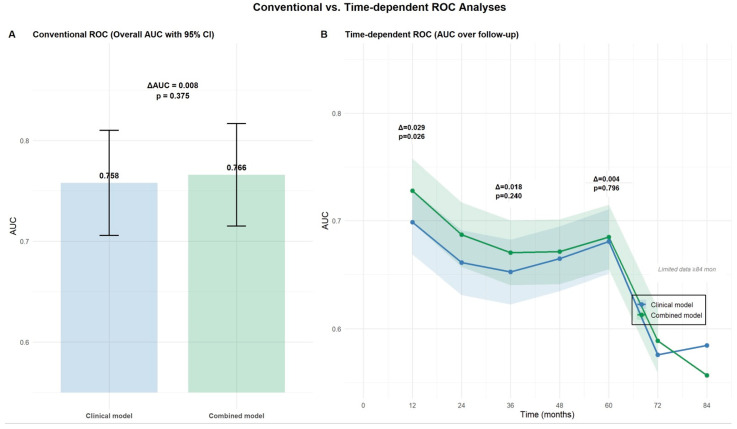
Conventional versus time-dependent ROC analyses of multivariable model and multivariable model plus AKI.

The time-dependent ROC analysis demonstrated that incorporating AKI into the multivariable clinical model, including age, COPD, valve Type (Balloon-expandable vs. Self-expandable), and pleural effusion, improved the model’s ability to predict all-cause mortality after TAVI, particularly during the early follow-up period. At 12 months, the combined model achieved a significantly higher AUC compared to the clinical model alone (ΔAUC = 0.029, *p* = 0.026, Figure 8). However, this predictive advantage diminished over time, with non-significant differences observed at 24 months (ΔAUC = 0.018, *p* = 0.240) and 60 months (ΔAUC = 0.004, *p* = 0.796). In contrast, the conventional ROC analysis showed no significant overall improvement with the addition of AKI (AUC: 0.766 vs. 0.758, ΔAUC = 0.008, *p* = 0.375, Figure 8).

## 5. Discussion

In this study, we demonstrated that AKI is a significant determinant of early and mid-term mortality following TAVI, with its prognostic effect being most pronounced during the first year after the procedure. The findings from landmark analyses and time-varying Cox regression consistently highlight the dynamic nature of AKI’s impact on survival. Specifically, AKI was associated with a markedly increased hazard of death in the early post-procedural period, which gradually attenuated during mid-term follow-up and eventually lost statistical significance beyond 12 months.

These results are in line with previous observational studies that identified AKI as a predictor of short-term mortality after TAVI [6,7,8,9]. Also, patients undergoing surgical aortic valve replacement (SAVR) exhibited a markedly increased risk of early postoperative AKI, which was associated with prolonged the intensive care unit (ICU) stay and significantly worse survival, particularly in those who required renal replacement therapy [13]. Acute renal impairment in this population may trigger adverse hemodynamic consequences, including fluid retention, electrolyte disturbances, and worsening cardiac function, thereby predisposing patients to early death [10]. In addition, AKI has been linked to systemic inflammatory responses and endothelial dysfunction, both of which may exacerbate the risk of cardiovascular events in the immediate post-procedural phase [14,15].

The attenuation of AKI’s prognostic effect in the long-term suggests that other factors gradually outweigh the initial impact of renal injury [16]. Patients who survive the early post-TAVI period may stabilize hemodynamically and recover partially from the acute renal insult, thereby reducing the relative contribution of AKI to mortality risk. Moreover, long-term outcomes after TAVI are increasingly influenced by factors such as prosthesis durability, progressive heart failure, arrhythmias, and non-cardiac comorbidities, which may dilute the prognostic weight of AKI over time [17,18].

From a clinical perspective, our findings underscore the importance of preventive strategies aimed at minimizing the risk of AKI in TAVI patients. This includes judicious use of contrast media, pre-procedural hydration, careful patient selection, and close monitoring of renal function in the immediate post-procedural phase [19,20]. In addition, recognition of AKI as a high-risk marker may inform closer follow-up and tailored management strategies, particularly within the first year after TAVI.

In the present study, the adverse prognostic impact of AKI on mortality remained consistent across all clinical subgroups, indicating that AKI represents a universally detrimental event following TAVI, regardless of baseline characteristics or procedural factors. Although a trend toward a stronger association was observed in patients with impaired renal function [21] and those receiving self-expandable valves, these findings did not reach statistical significance, suggesting that the mortality risk conferred by AKI is broadly applicable rather than confined to specific patient subsets.

Finally, the application of landmark and time-varying analyses provides important methodological insights. Traditional Cox models assume proportional hazards, which may not adequately capture the evolving nature of AKI’s risk profile. By contrast, the approaches we used revealed a more nuanced temporal pattern, demonstrating the highest risk early after the procedure and a decline thereafter [22,23,24,25,26]. These methodological considerations should be incorporated into future prognostic studies in structural heart interventions.

This study has certain limitations that require recognition. The retrospective, two-center design inherently introduces selection bias and limits the generalizability of the results across heterogeneous TAVI populations with differing demographic and procedural characteristics [27,28,29]. Even with the application of rigorous inclusion and exclusion criteria, residual confounding from unmeasured variables—such as hydration status, contrast volume, intra-procedural hemodynamic variability, or minor variations in baseline renal function—cannot be completely eliminated. Secondly, AKI was defined based on conventional creatinine-based criteria, which may inadequately reflect temporary or subclinical renal impairment; furthermore, we did not evaluate emerging renal biomarkers such as NGAL or cystatin-C, which could identify earlier tubular damage [30]. Third, the volume and type of contrast agent were not consistently standardized among all procedures and operators, and detailed information regarding periprocedural nephroprotective techniques (e.g., type and rate of hydration, administration of N-acetylcysteine, or sodium bicarbonate) was absent in some cases [31]. Fourth, although we analyzed long-term all-cause mortality, we could not distinguish between cardiac and non-cardiac causes of death, which may obscure the precise impact of AKI on cardiovascular outcomes. Fifth, follow-up data were sourced from hospital records and national databases, which may lead to incomplete outcome ascertainment, especially for individuals treated at different institutions. Ultimately, this was a retrospective analysis; therefore, despite multivariable adjustment, the possibility of residual confounding cannot be completely excluded. Patients who developed AKI may have had unmeasured baseline differences or higher underlying clinical frailty that were not fully captured by the available variables. Although AKI remained an independent predictor of long-term mortality in the adjusted Cox model, causality cannot be definitively inferred, and the association should be interpreted in the context of potential unmeasured confounders. Further prospective multicenter trials are necessary to confirm the prognostic significance of contrast-induced nephropathy after TAVI. 

## 6. Conclusions

AKI following TAVI was associated with significantly increased early and mid-term mortality, particularly within the first month and first year after the procedure. However, the impact of AKI on long-term outcomes appeared to diminish beyond the first year, suggesting that the prognostic significance of post-procedural renal dysfunction is time-dependent and may be influenced by subsequent renal recovery or competing non-renal risk factors.

## Figures and Tables

**Table 1:** Baseline Characteristics of Patients With and Without Acute Kidney Injury (AKI).

Variable	No AKI (n = 322)	AKI (n = 59)	*p*
Age (years)	77.21 ± 7.24	77.29 ± 7.09	0.942
Gender (Male)	163 (51%)	21 (36%)	0.034
Diabetes Mellitus	107 (33%)	24 (41%)	0.268
Hypertension	204 (63%)	40 (68%)	0.513
Coronary Artery Disease	244 (76%)	45 (76%)	0.935
Peripheral Artery Disease	44 (14%)	9 (15%)	0.746
Heart Failure	66 (20%)	13 (22%)	0.789
COPD	22 (6.8%)	6 (10%)	0.366
Previous Stroke	20 (6.2%)	2 (3.4%)	0.393
Atrial Fibrillation	88 (27%)	14 (24%)	0.566
In-Hospital Mortality	6 (1.9%)	7 (12%)	0.001
All-Cause Mortality	78 (24%)	28 (47%)	<0.001
Aortic Peak Gradient (mmHg)	78.04 ± 22.53	73.26 ± 21.78	0.136
Aortic Mean Gradient (mmHg)	47.27 ± 14.60	43.68 ± 13.43	0.080
Moderate to severe mitral regurgitation (%)	119 (37%)	30 (51%)	0.044
Moderate to severe tricuspid regurgitation n (%)	81 (25%)	24 (41%)	0.014
Valve Type n (%)			0.095
Self expandable	198 (61%)	43 (73%)	
Baloon expandable	124 (39%)	16 (27%)	
Pleural Effusion	114 (35%)	33 (56%)	0.003
Red cell transfusion n (%)	28 (9)	11 (19)	0.020
Cardiac tamponade n (%)	3 (1)	2 (3)	0.127
Vascular complications n (%)			0.012
Pseudoaneurysm	3 (1)	3 (5)	
Arterial injury treated with stent	9 (3)	5 (9)	
Arterial injury treated with surgery	13 (4)	1 (2)	
Chronic kidney disease n (%) *			0.060
Stage 1	58 (18)	5 (9)	
Stage 2	157 (49)	26 (44)	
Stage 3a	56 (17)	10 (17)	
Stage 3b	44 (14)	15 (25)	
Stage 4	7 (2)	3 (5)	
LVEF (%)	54.69 ± 10.50	53.25 ± 11.84	0.344
Platelet Count (10^3^/µL)	218.57 ± 84.87	218.83 ± 74.63	0.983
White blood cell counts (10^3^/µL)	7.86 ± 3.3	8.18 ± 2.8	0.484
Hemoglobin (g/dL)	11.25 ± 1.73	10.89 ± 1.86	0.116
eGFR (mL/min/1.73 m^2^)	68.87 ± 20.34	60.14 ± 19.83	0.003
Creatinine (mg/dL) at admission	1.04 ± 0.36	1.15 ± 0.38	0.039
Creatinine (mg/dL) at 48–72 h	1.01 ± 0.32	1.99 ±0.95	< 0.001

Abbreviations: COPD; Chronic Obstructive Pulmonary Disease, LVEF; Left ventricular ejection fraction, eGFR; estimated glomerular filtration rate. * Since patients with eGFR (mL/min/1.73 m^2^) <20 were excluded from the study, there were no patients in stage 5.

**Table 2:** Baseline Characteristics of Survivors and Non-Survivors After TAVI.

Variable	Survivors (275)	Non-Survivors (106)	*p*
Age (years)	76.50 ± 7.10	79.11 ± 7.18	0.001
Gender (Male) n (%)	133 (48%)	51 (48%)	0.965
Diabetes Mellitus n (%)	97 (35%)	34 (32%)	0.566
Hypertension n (%)	179 (65%)	65 (61%)	0.492
Coronary Artery Disease n (%)	203 (74%)	86 (81%)	0.135
Peripheral Artery Disease n (%)	33 (12%)	20 (19%)	0.083
Heart Failure n (%)	56 (20%)	23 (22%)	0.773
COPD n (%)	14 (5.1%)	14 (13%)	0.007
Previous Stroke n (%)	18 (6.5%)	4 (3.8%)	0.299
Atrial Fibrillation n (%)	68 (25%)	34 (32%)	0.147
In-Hospital Mortality n (%)	0 (0%)	13 (12%)	< 0.001
Aortic Peak Gradient (mmHg)	76.97 ± 22.37	78.20 ± 22.75	0.634
Aortic Mean Gradient (mmHg)	46.84 ± 14.65	46.35 ± 14.04	0.768
Moderate to severe mitral regurgitation n (%)	95 (35%)	54 (51%)	0.003
Moderate to severe tricuspid regurgitation n (%)	68 (25%)	37 (35%)	0.046
Valve Type n (%)			<0.001
Self expandable	149 (54%)	92 (87%)	
Baloon expandable	126 (46%)	14 (13%)	
Pleural Effusion n (%)	90 (33%)	57 (54%)	<0.001
Red cell transfusion n (%)	24 (9)	15 (14)	0.118
Cardiac tamponade n (%)	3 (1)	2 (2)	0.541
Vascular complications n (%)			0.147
Pseudoaneurysm	3 (1)	3 (3)	
Arterial injury treated with stent	7 (3)	7 (7)	
Arterial injury treated with surgery	11 (4)	3 (3)	
Chronic kidney disease n (%) *			<0.001
Stage 1	44 (16)	19 (18)	
Stage 2	138 (50)	45 (43)	
Stage 3a	55 (20)	11 (10)	
Stage 3b	35 (13)	24 (23)	
Stage 4	3 (1)	7 (7)	
Acute Kidney Injury *n* (%)	31 (11%)	28 (26%)	<0.001
LVEF (%)	54.72 ± 10.82	53.83 ± 10.45	0.470
Platelet Count (10^3^/µL)	221.67 ± 82.00	210.70 ± 86.37	0.250
White blood cell counts (10^3^/µL)	7.92 ± 3.3	7.86 ± 2.9	0.854
Hemoglobin (g/dL)	11.35 ± 1.71	10.87 ± 1.87	0.016
eGFR (mL/min/1.73 m^2^)	68.91 ± 19.35	63.90 ± 22.86	0.032
Creatinine (mg/dL) at admission	1.03 ± 0.32	1.14 ± 0.46	0.010
Creatinine (mg/dL) at 48–72 h	1.06 ± 0.37	1.43 ± 0.89	<0.001

Abbreviations: COPD; Chronic Obstructive Pulmonary Disease, LVEF; Left ventricular ejection fraction, eGFR; estimated glomerular filtration rate. * Since patients with eGFR (mL/min/1.73 m^2^) <20 were excluded from the study, there were no patients in stage 5.

**Table 3:** Cox regression analysis for long-term all-cause mortality.

	Univariate Analysis		Multivariate Analysis	
Variable	HR (95% CI)	*p*	HR (95% CI)	*p*
Age (years)	1.04 (1.01–1.08)	0.011	1.03 (1.00–1.07)	0.044
Peripheral Artery Disease	1.37 (0.84–2.24)	0.202	—	—
Chronic Obstructive Pulmonary Disease	2.82 (1.60–4.98)	<0.001	3.34 (1.83–6.10)	<0.001
Hemoglobin (g/dL)	0.87 (0.77–0.97)	0.017	0.99 (0.88–1.11)	0.842
eGFR (mL/min/1.73 m^2^)	0.99 (0.98–1.00)	0.035	0.99 (0.98–1.00)	0.247
Moderate to Severe Mitral Regurgitation	1.68 (1.15–2.46)	0.008	1.15 (0.75–1.78)	0.516
Moderate to Severe Tricuspid Regurgitation	1.50 (1.01–2.24)	0.047	0.95 (0.60–1.51)	0.840
Valve Type (Balloon-expandable vs. Self-expandable)	0.31 (0.18–0.54)	<0.001	0.32 (0.18–0.58)	<0.001
Pleural Effusion	2.01 (1.37–2.94)	<0.001	1.56 (1.03–2.36)	0.035
Acute Kidney Injury	2.46 (1.59–3.78)	<0.001	2.07 (1.35–3.25)	0.002

Abbreviations: eGFR, estimated glomerular filtration rate.

## Data Availability

The data presented in this study are available on request from the corresponding author due to privacy concerns.
